# Standard Survey Data: Insights Into Private Sector Utilization

**DOI:** 10.3389/fmed.2021.624285

**Published:** 2021-04-12

**Authors:** Dominic Montagu, Nirali Chakraborty

**Affiliations:** ^1^Department of Epidemiology and Biostatistics, University of California, San Francisco, San Francisco, CA, United States; ^2^Metrics for Management, Baltimore, MD, United States

**Keywords:** private sector, health seeking behavior, universal health coverage, health policy, health governance

## Abstract

Universal Health Coverage in Low- and Middle-Income Countries is increasingly expanding through incorporation of private clinics, pharmacies, and hospitals into an overall health system funded in whole or part through government-managed health insurance. This underscores the importance of policies on health provision which apply across the whole delivery system regardless of ownership status. To advance understanding of private-sector policies, and to facilitate sharing of lessons across countries with similar public-private distributions, we have analyzed data on the source of inpatient and outpatient care from 65 countries. While past studies have conducted similar analysis, ours advances the field in two ways. First, we limit our analysis to data sets from 2010 through 2019, making our study more up-to-date than past studies, while changing health seeking patterns for maternal health since 2010 means that our data set is more representative of overall inpatient care. Second, while past multi-country analysis of public-private ownership have been based on the Demographic Health Surveys, we have added to this data from the Multiple Indicator Cluster Surveys, significantly increasing the countries in our analysis. We have aggregated our analysis by WHO's regions. Outside of the EURO region, where the private sector delivers just 4% of all healthcare services, the private sector remains significant, and in many countries represents more than half of all care. The private sector provides nearly 40% of all healthcare in PAHO, AFRO, and WPRO regions, 57% in SEARO, and 62% in EMRO. While specific countries with two recent surveys show variation in the scale of both inpatient and outpatient private provision, we did not find regional or global trends toward or away from private care within LMICs. Private inpatient care is most important for the wealthy in many countries; public vs. private care varies less, by wealth, for outpatient services.

## Introduction

This study sets out to summarize the importance of private provision of inpatient and outpatient care within health systems of 65 countries in Latin America, Africa, Europe, and Asia. We expand on and update prior studies which have used similar data, and create regional summaries. The reason to undertake this analysis is the change in delivery patterns around the world which makes our data set of inpatient source of care a better proxy for overall inpatient care than was true in the past, and the expansion in recent years of both the system level goal of Universal Health Coverage (UHC) and of the use of Social Health Insurance (SHI) as a vehicle to advance it (1). SHI initiatives are increasingly engaging the private sector as a necessary way to achieve UHC in countries where a large part of the existing service provision infrastructure, providers, and care-seeking, is private.

The push toward UHC has thus underscored the need to assure common standards of care are applied to both public and private sector facilities and providers, and that all sources of healthcare are coordinated regardless of ownership status. Referrals between public and private providers, pharmacies, laboratories, blood-banks, and hospitals must be seamless and efficient if care for patients is to take precedence over monopolies driven either by profit or ideology. Sharing of data, diagnosis, medicines, and information is often critical for public health. The experiences of tuberculosis and vaccines have shown the world that integration of care between public and private is possible, and that when done well it can greatly advance health goals (2). Advancing both integration and common standards requires adjustments, often significant adjustments, to regulatory systems designed to address only the public sector. These changes include policy, programmatic, and implementation challenges (3). Addressing them requires the awareness and attention of policy makers, and of global institutions which can provide guidance and examples of relevant best practices. The work of this study is intended to inform both of these constituencies, as well as providing information on where lessons applicable to any one country may best be drawn.

### Measuring the Private Sector

For nearly 20 years nationally representative surveys have been used to identify variations in access to health services by country and region as well as across wealth and geographic regions within countries. Using Demographic and Health Surveys (DHS) researchers have been able to inform policy and program decision-making with information on care-seeking for families with pediatric illnesses, maternity services, and family planning. This survey data can inform country comparisons. It matters, for example, for national governments, international agencies, and donors to know that 80% of pediatric care in Pakistan is sourced exclusively from the private sector, while in Ethiopia the percent of all pediatric care that is private is only 24% (4, 5).

Payment data has the potential to offer an alternative measure of public-private health mix in many countries, however there are challenges. With some variation depending on the source data used, there is consistency in findings that 95% or more of all private expenditure on health is out-of-pocket (OOP) payment for care directly to providers, and that OOP payments are surprisingly stable, hovering around two percent of GDP. An important implication of this is that OOP decreases as a percent of total healthcare expenditures as countries become wealthier and government expenditures toward health increase (6). Domestic private expenditure makes up nearly 50% of current health expenditure in the WHO AFRO region, 61.4% in EMRO and 67.5% in SEARO (7). While private voluntary insurance and social health insurance expansion is changing this slowly, the effects of both are relatively small in LMICs (1, 8).

Expenditures are a strong measure of the risks of household impoverishment and of access challenges, but show poor correlation to source of care: payments are made to both public and private providers. As such, OOP payments are not a good measure of the importance of the private sector relative to overall healthcare service provision (6, 9, 10). Utilization of care, measured through mostly-standardized questions on large-scale household surveys around the world, provides a more stable and comparable—across times and countries—estimate of the private sector's importance overall. This in turn can inform the need for policy attention to focus specifically on privately owned pharmacies, clinics, and hospitals within the context of overall health systems regulation and guidance.

The importance of policy decisions specific to private healthcare provision has been underscored by recent analysis (10), and by the growing recognition that private healthcare, in many Low- and Middle-Income Countries, is external to both the regulatory and financing systems which are expanding to assure Universal Health Coverage (3, 11). Where regulatory and subsidy systems have included private providers, the results have been overall improvements in access and quality; better than when these same providers act externally to the national overall health regulatory structure (12). Evidence on the importance of private provision within overall national contexts is therefore important as policy makers consider how much attention to give to this issue. Informing this is knowledge of what other countries might have provider sectors of roughly equivalent scale and so provide models worth examining to inform national policies (13–16).

Past efforts to provide this evidence have relied primarily on the DHS as the sole source of nationally representative data on source of care (4, 17–19). We have both updated the DHS data used from prior studies, and nearly doubled our data points by added in surveys from UNICEF's Multiple Indicator Cluster Surveys (20).

Our analysis examines 65 countries using DHS and MICS surveys, including more countries than previous studies by Campbell and Footman (21–23). Our work also utilizes data sets from 2010 to 2019, updating previous work that used data from time points between 1990 and 2014 (18, 21–25). We have used a specific definition of the private sector to include private hospitals, NGO or faith-based hospitals, private clinic/doctor, private pharmacy, and other NGO or faith-based operations such as clinics, outreach services, or community health workers. Our specificity of the definition of the private sector, examination of changes over time, and use of recent data sources are novel.

## Methods

Using freely available standardized, nationally representative survey data, we estimate the relative use of the public and private sectors across a diverse set of Low- and Middle-Income Countries. After reviewing available data sources, only the Demographic and Health Surveys (DHS) and the Multiple Indicator Cluster Surveys (MICS) provided comparable and comprehensive information on source of care for inpatient and outpatient conditions.

### Data

Parameters for inclusion were that a country has a nationally representative MICS or DHS survey between 2014 and 2019. A second survey, if present, needed to have been conducted between 2010 to within 3 years of the first survey, with preference given for the shortest time period within this if there are more than a single survey meeting these criteria. Surveys spanning 2 years (such as 2014–2015) are defined as having been conducted in the earlier year, which has implications for inclusion criteria in some instances. One hundred and twelve surveys met these criteria, however one survey was excluded because the data did not match the other datafiles (Guinea-Bissau MICS 2010). The total number of countries included in the analysis is 65.

The analysis uses information on care seeking for delivery as a proxy for inpatient care. Similarly, care seeking for childhood illnesses is a proxy for outpatient care. The analysis investigates proportion of care sought within the public vs. private sector. Surveys conducted between 2010 and 2019 cover DHS rounds 6 and 7, and MICS rounds 4–6. The surveys differ across rounds, in definition of illness, and populations for whom data is collected. For example, in DHS surveys, the analysis obtains information on the place of birth, for a woman's most recent birth in the past 5 years, while in MICS surveys, this may be in the past 2 or 3 years. For childhood illnesses, the analysis categorizes the place of care sought, for an illness in the prior 2 weeks for the youngest child under age 5 in the household. Illnesses included in the study are diarrhea, or Acute Respiratory Infection (ARI)/Fever. Care seeking for ARI (suspected pneumonia) and fever are reported together in all of the surveys for which children with fever are asked about place of care sought.

### Definitions

The definition of ARI used is consistent with that used for each source survey, and the definitions differ across source surveys (DHS 6, DHS 7, MICS6, MICS 4, and 5).

#### Definitions of ARI

##### DHS 6

Cough accompanied by short rapid breathing. Children with cough who do not meet definition of ARI were still asked about care-seeking. We have removed them from the denominator and only conducted analyses on those who meet definition of fever or ARI (suspected pneumonia) as defined by the survey.

##### DHS 7

Short rapid breathing which was chest-related, and/or difficult breathing which was chest related. Children with rapid breathing that is not chest related do not meet the definition of ARI, but were still asked about care-seeking. We have removed them from the denominator and only conducted analyses on those who meet definition of fever or ARI (suspected pneumonia) as defined by the survey.

##### MICS (4–6)

Definition of ARI is illness with a cough, accompanied by a rapid or difficult breathing and whose symptoms were due to a problem in the chest, or both a problem in the chest and a blocked nose. Children with rapid breathing that is not chest related do not meet the definition of ARI, but were still asked about care-seeking. We have removed them from the denominator and only conducted analyses on those who meet definition of fever or ARI (suspected pneumonia) as defined by the survey. In some MICS5 surveys, the question on whether the problem was in the chest is not asked. In MICS4 surveys, place of care-seeking for fever or diarrhea is not asked.

For each household with an included reason for care, the analytic dataset captures the country, year, household weight, household wealth quintile, reason for needed care (recent birth, diarrhea, fever or ARI) and source(s) of care for the illness. Additionally, we include the WHO region and sub-region, and country population for the year of the survey from the UN Population Division. For childhood illnesses, more than one source of care for an episode was possible. Source of care was manually recategorized into one of 9 mutually exclusive categories (Box 1). Each country's data extraction and log files were rechecked by a second analyst for quality control. Surveys from Cuba and Qatar did not include any wealth quintiles. Surveys from Columbia 2015, Serbia and Kazakhstan did not ask about childhood illness.

Box 1 **Sources of care**

**1. Public Sector**

a. Hospital
  i. Classify hospital/clinic as a hospital
b. Everything else
  i. Clinic, CHW, PHC, etc

**2. Private sector**

a. Private Hospital
b. NGO or FBO Hospital
c. Private Clinic / doctor
d. Private Pharmacy
e. NGO or FBO other
  *i. NGO clinic, NGO outreach, NGO Community health worker, etc*.
f. Shop, drug seller, other (classified as informal)

**3. No care sought outside of home**

If it was not clear what sector a source of care belongs to, then it is classified as “other.” As a result, some care that is counted as informal may actually be provided by a trained health worker. An example of this is a code of “fieldworker,” without any specification as to what sector the fieldworker belongs to. In some analyses, private sector care is sub-categorized into (a) Private hospitals, clinics, doctors, and pharmacies; (b) NGO and FBO facilities; (c) Informal facilities.

##### Regional Analyses

For the most recent survey for countries within each WHO region, data were weighted by country population size for the year of the survey. Wealth quintiles were kept as in the original country data, so the regional analyses by wealth represent the behavior of households in the same relative wealth groups.

##### Assumptions and Limitations

We make a number of assumptions in summarizing and analyzing this data, and in particular in drawing conclusions about all inpatient, outpatient, and overall health system usage based on sources for only a few care-seeking practices. We do so primarily because these data provide more information with which to make system-level inferences than other sources. We use them not for their accuracy, but because they are less inaccurate than other options.

Specifically, in our analysis we assume that care seeking patterns for childbirth represent inpatient care seeking patterns. We assume that care seeking for routine childhood illnesses (ARI, Fever and Diarrhea) represent outpatient care seeking patterns. We recognize that these assumptions are weak, and that they are simply a result of lack of available data across countries for care sought for other reasons. Additionally, the data on childhood illness is with regard to illnesses in the 2 weeks prior to the survey, while for birth, the recall period ranges from 2 to 5 years, depending on the survey. No attempt was made to reconcile the time periods, when describing care-seeking patterns as the focus of this analysis is on ratios of care-seeking, not quantity of care-seeking. WHO analysis shows that of all spending on health within 46 LMIC for which there is data, 25% is spent on inpatient and day curative care, and 28% is spent on outpatient and home-based curative care (7). Using this ratio, we weight the data on source of care by reason, in order to derive an estimate of overall care-seeking patterns.

## Results

Of the 65 countries for which we have data, the majority are in the WHO African region (26), followed by the region of the Americas (11). There were 49 countries with MICS surveys meeting inclusion criteria, and 62 countries with DHS surveys. (See Table 1 for list).

**Table 1:** Country and data sources included.

	**Survey type**	
**Country**	**MICS**	**DHS**
Afghanistan	1	1
Albania		1
Angola		1
Armenia		2
Bangladesh		2
Belize	2	
Benin	1	1
Burundi		2
Cambodia		2
Cameroon	1	1
Chad	1	1
Colombia		2
Congo	1	1
Cote d'Ivoire	1	1
Cuba	2	
Dominican Republic	1	
Egypt		1
El Salvador	1	
Eswatini	2	
Ethiopia		2
Gambia	1	1
Ghana	1	1
Guatemala		1
Guinea		2
Guinea-Bissau	1	
Guyana	1	
Haiti		2
India		1
Indonesia		2
Iraq	2	
Jordan		2
Kazakhstan	2	
Kenya		1
Kyrgyzstan	2	
Lao People's Democratic Republic	2	
Lesotho		1
Malawi		2
Maldives		1
Mali	1	1
Mauritania	2	
Mexico	1	
Mongolia	2	
Myanmar		1
Nepal		2
Nigeria	1	1
Pakistan		2
Paraguay	1	
Philippines		2
Rwanda		2
Sao Tome and Principe	1	
Senegal^*^		2
Serbia	2	
Sierra Leone	1	1
South Africa		1
State of Palestine	2	
Sudan	2	
Suriname	2	
Tajikistan		2
Tanzania		2
Thailand	2	
Timor-Leste		1
Tunisia	2	
Turkmenistan	1	
Uganda		2
Zimbabwe		2
**Total**	**49**	**62**

* *Senegal uses a continuous DHS survey*.

**Figure 1:**
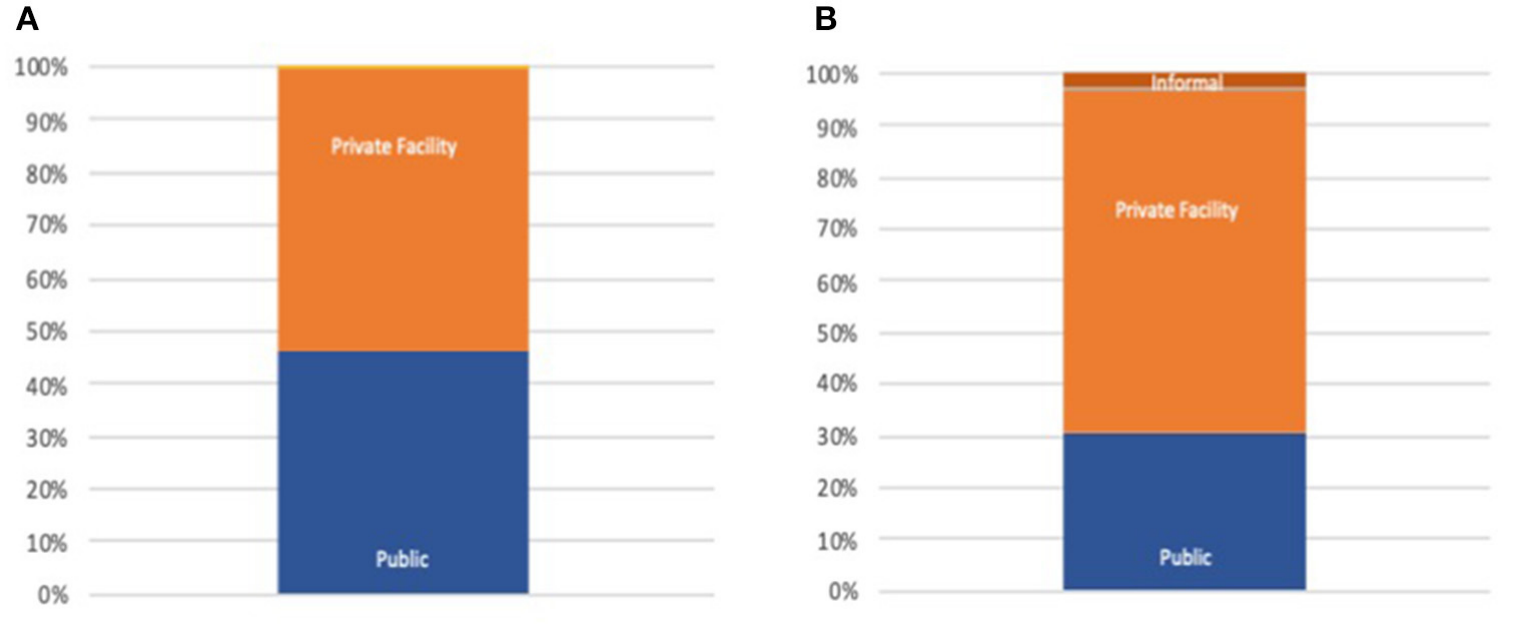
**(A)** Public/Private inpatient distribution EMRO. **(B)** Public/Private outpatient distribution EMRO.

The region with the greatest reliance on the private sector is the Eastern Mediterranean region; weighted regional results from most recent surveys in each country indicate that 53% of inpatient and 66% of outpatient care takes place in the for-profit private sector (Figure 1). This data is heavily influenced by Egypt and Pakistan. Conversely, citizens in the WHO European region are the most reliant upon public sector services, as seen within the eastern European and Central Asian countries for which we have data (96% of care sought in public sector).

**Figure 2:**
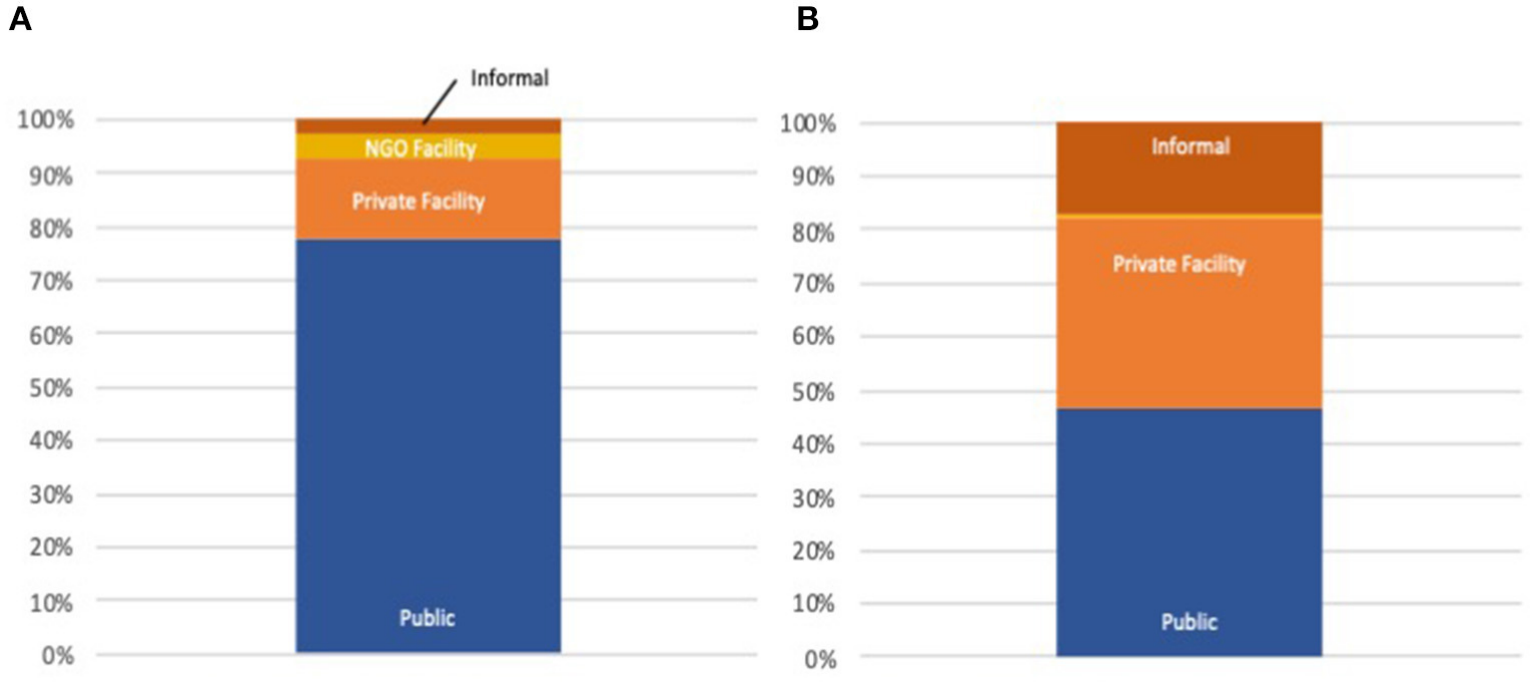
**(A)** Public/Private inpatient distribution AFRO. **(B)** Public/Private outpatient distribution AFRO.

For outpatient care in Africa, 35% of those who seek care go to the for-profit private sector, while 17% seek care at shops, faith healers and other informal providers (Figure 2). Overall, 26% of care seeking is done in the private sector, with an additional 10% with informal providers. The greatest proportion of private sector care seeking occurs in Nigeria (52%), while in Cameroon, Uganda and Benin, >40% of care is sought in the private sector. All figures are for those who choose to seek care for the conditions studied in this analysis.

Table 2 lists the top three most privatized countries in each WHO region, and the overall, inpatient and outpatient proportion of care sought in for-profit private sector (excluding NGO or Informal). Table 3 gives ownership proportions for each WHO region.

**Table 2:** Proportion of care provided by private sector medical providers—top 3 countries per region.

		**Private as a percent of all care**		
**Region**	**Country**	**Total**	**Inpatient**	**Outpatient**
African	Uganda	40.2	21.4	56.9
	Nigeria	36.8	33	40.2
	Swaziland	29	31.6	26.7
Americas	Mexico	33	15.8	48.3
	Suriname	28.6	29.9	27.4
	Dominican Republic	28.3	29.7	27.1
Eastern Med	Egypt	75.2	71.2	78.8
	Pakistan	73.8	65.5	81.2
	Jordan	44.9	33.9	54.6
European	Albania	7.8	3.4	11.8
	Kyrgyzstan	7.1	1.5	12.1
	Armenia	4.5	3.8	5.4
South-East Asia	Indonesia	60	61	59.1
	Bangladesh	57.2	59.5	55.2
	India	52.6	34.3	68.8
Western Pacific	Cambodia	33	17.6	46.7
	Philippines	32.2	29.1	34.9
	Laos	14.6	2.5	25.3

**Table 3:** Ownership ratio by WHO region.

		**Inpatient (%)**	**Outpatient (%)**
PAHO	Informal	1	9
	NGO	0	0
	Private	31	37
	Public	68	54
AFRO	Informal	3	17
	NGO	4	1
	Private	15	35
	Public	78	47
EMRO	Informal	0	3
	NGO	0	0
	Private	53	66
	Public	46	31
EURO	Informal	0	1
	NGO	0	0
	Private	1	7
	Public	99	93
SEARO	Informal	0	7
	NGO	1	0
	Private	35	68
	Public	64	24
WIPRO	Informal	3	12
	NGO	0	0
	Private	27	36
	Public	70	51

*Weighted by country population from year of most recent DHS or MICS survey*.

For those countries with more than one data set we have examined the changes in percent of care sourced from private providers (Table 4). While variations are clearly smaller for inpatient services than outpatient (reflecting perhaps the stability inherent to high-infrastructure investment costs), there are no clear global or regional trends toward overall increase or decrease of private care.

**Table 4:** Change in private sector percentage of service provision, most recent two surveys.

**Country**	**Inpatient (%)**	**Outpatient (%)**
Afghanistan	1	−8
Armenia	0	0
Bangladesh	0	−2
Belize	1	8
Benin	1	−8
Burundi	−2	−2
Cambodia	−1	−1
Cameroon	0	−18
Chad	−2	9
Colombia	0	0
Congo	1	3
CoteD'Ivoire	3	−15
Cuba	0	0
Ethiopia	1	1
Gambia	1	1
Ghana	−1	−9
Guinea	0	10
Haiti	0	−1
Indonesia	0	1
Iraq	0	−1
Jordan	1	0
Kazakhstan	0	0
Kyrgyzstan	0	−1
Laos	−5	4
Malawi	−2	−4
Mali	−1	5
Mauritania	0	13
Mongolia	0	0
Nepal	−2	−1
Nigeria	5	12
Pakistan	0	−1
Palestine	−1	−16
Philippines	2	0
Rwanda	−1	−8
Senegal	2	−5
Serbia	0	0
SierraLeone	0	3
Sudan	−6	10
Suriname	3	−7
Swaziland	−1	3
Tajikistan	0	−1
Tanzania	0	2
Thailand	0	1
Tunisia	0	−2
Uganda	0	−3
Zimbabwe	1	2

## Discussion

We are making inferences regarding overall public/private healthcare which have significant implications for policy attention to private providers, based upon a limited set of data. This is due to what data is available, rather than what data would be ideal, but we have no reason to believe there is a systematic bias to the conclusions we draw.

### Drawing Conclusions From Imperfect Data

Perhaps most notably, we use self-reported information on delivery location as a proxy for overall public-private inpatient care ratios. We do not make adjustments for potential differences in care seeking decision differences for delivery vs. other inpatient care seeking; nor do we adjust for differences in bed turnover rates between public and private facilities. We do not explore if delivery rates are reflective of staffing, costs, and health outcomes. These are all important areas for future exploration.

A comparison of OECD reported data and DHS data from Mexico shows that all-bed ratios and patient reported delivery site ratios are broadly aligned; 26% private for all-beds, vs. 16% private for deliveries (27). Data from Kenya suggests that private bed turnover ratios (the number of deliveries/bed) are roughly half that of public facilities, meaning that for every 10 women who report delivering in a private facility 20 will have delivered in a public facility of the same size (26). A study from Nepal suggests that bed occupancy rates in Maternity wards is not very different from overall hospital bed occupancy rates (91 vs. 74%) and, importantly for our study, that more than two fifths of all inpatients (41.86%) were admitted in the maternity ward (28). Facility deliveries have increased significantly in the past decade (24). With the exception of only the poorest quintiles in the AFRO region, the majority of the respondents included in our analysis delivered their last child in a healthcare facility. Based on all of this we believe that in the absence of better data it is appropriate to use place-of-delivery data as an unbiased proxy for overall inpatient care in LMICs around the world. A similar argument justifies the use of our pediatric data as a proxy for outpatient care sources.

## Conclusions

Our findings confirm earlier studies showing that the private sector remains dominant for outpatient care in many countries, particularly in AFRO, EMRO, and SEARO regions, and significant in inpatient care across the same parts of the world. Comparing our findings to earlier studies, and across repeated surveys within our timeframe, we do not find any clear trend to increasing or decreasing private provision as a component of LMIC health systems. This is perhaps not surprising: the evidence from health systems in OECD countries shows a wide range of ownership frameworks associated with country delivery models, no one clearly evident of better quality or efficiency than another (29, 30). Indeed, in a number of countries the ratio of public to private ownership of care delivery have changed significantly within just a few years as policies have shifted: evidence of the political nature of ownership decisions, but not of the benefits of one ownership model over another which, if clear, would lead to a trend as countries advance in wealth or effectiveness of national governance (31, 32). Rather than search for, plan for, or encourage a shift toward greater or smaller private participation in healthcare deliver, our findings show that mixed healthcare systems remain the norm in LMIC countries, across regions, and across wealth levels within countries.

The implications of this for UHC are important for regulation and policy, and the national planning bodies responsible for governance together with the global agencies that advise and support them: the management of mixed public and private healthcare systems will determine the success or failure to achieve UHC for many countries. Our analysis should provide countries with a path to identify nations with similar levels of public-private mix, with which to study and share experiences on quality assurance, reporting, referral integration, financing systems, and the many other aspects critical to good management in a complex delivery context.

Further work and examination of lessons specific to countries and regions will be needed to inform better policies in the future.

## Data Availability Statement

Publicly available datasets were analyzed in this study. This data can be found here: https://dhsprogram.com/; https://mics.unicef.org/.

## Author Contributions

DM and NC designed the study, agreed on interpretation, and analysis of results. NC extracted the data, led the analysis, and reviewed and edited the final draft. DM wrote the paper. Both authors contributed to the article and approved the submitted version.

## Conflict of Interest

The authors declare that the research was conducted in the absence of any commercial or financial relationships that could be construed as a potential conflict of interest.
